# Phospholipase D2 Enhances Epidermal Growth Factor-Induced Akt Activation in EL4 Lymphoma Cells 

**DOI:** 10.3390/ph3072045

**Published:** 2010-07-02

**Authors:** Manpreet S. Chahal, Daniel J. Brauner, Kathryn E. Meier

**Affiliations:** 1 Department of Pharmaceutical Sciences, College of Pharmacy, Washington State University, Pullman, Washington, WA 99164-6534, USA; E-Mail: manpreet.chahal@email.wsu.edu (M.C.);; 2 Program in Nutrition and Exercise Physiology, College of Pharmacy, Washington State University, Spokane, Washington, WA99210-1495, USA

**Keywords:** protein kinase inhibitors, phospholipids metabolism, metastasis

## Abstract

Phospholipase D2 (PLD2) generates phosphatidic acid through hydrolysis of phosphatidylcholine. PLD2 has been shown to play a role in enhancing tumorigenesis. The epidermal growth factor receptor (EGFR) can both activate and interact with PLD2. Murine lymphoma EL4 cells lacking endogenous PLD2 present a unique model to elucidate the role of PLD2 in signal transduction. In the current study, we investigated effects of PLD2 on EGF response. Western blotting and RT-PCR were used to establish that both parental cells and PLD2 transfectants express endogenous EGFR. Levels of EGFR protein are increased in cells expressing active PLD2, as compared to parental cells or cells expressing inactive PLD2. EGF stimulates proliferation of EL4 cells transfected with active PLD2, but not parental cells or cells transfected with inactive PLD2. EGF-mediated proliferation in cells expressing active PLD2 is dependent on the activities of both the EGFR and the PI3K/Akt pathway, as demonstrated by studies using protein kinase inhibitors. EGF-induced invasion through a synthetic extracellular matrix is enhanced in cells expressing active PLD2, as compared to parental cells or cells expressing inactive PLD2. Taken together, the data suggest that PLD2 acts in concert with EGFR to enhance mitogenesis and invasion in lymphoma cells.

## 1. Introduction

Phospholipase D (PLD) catalyzes the hydrolysis of phosphatidylcholine (PC) to produce phosphatidic acid (PA), a lipid second messenger [1,2]. There are two mammalian PLD isoforms, PLD1 and PLD2. PLD2 expression has been detected in many organisms and in most mammalian tissues and cell types [3]. PLD2 activity is regulated by lipids, kinases, and small GTP-binding proteins [4,5]. Following agonist stimulation, PLD2 can redistribute within the cell [4,6,7]. PLD2-generated PA modulates signaling either by binding to proteins directly, or through its metabolites [8]. PA interacting proteins include sphingosine 1-phosphate kinase, Raf-1, cAMP-specific phosphodiesterase, mammalian target of rapamycin (mTOR), p70S6K, and phospholipase C-g1 [8,9,10,11]. PA can be converted to the lipid mediators diacylglyerol or lysophosphatidic acid, thereby activating additional signaling pathways [12,13]. PLD2 can also affect signaling pathways by binding directly to other proteins [14].

PLD2 regulates various cellular processes such as proliferation, adhesion, survival, apoptosis and tumor transformation [1,2,4,15]. Growth factors (e.g., FGF, EGF, insulin) generally stimulate PLD2 activity [16]. Increased PLD2 expression contributes to the transformation of fibroblasts overexpressing tyrosine kinases, such as c-Src or epidermal growth factor receptor (EGFR) [17]. Elevated PLD2 expression inhibits apoptotic signals via a p53-independent pathway in rat fibroblasts and breast cancer cells, while promoting survival signals through the mTOR pathway [18,19]. PLD2 bypasses cell cycle arrest programs that are often activated by apoptotic pathways, leading to tumor progression [19,20]. In v-Src transformed rat fibroblasts, overexpression of PLD2 significantly increases the size of cell protrusions, while opposite effects (elimination of cell protrusions) were observed upon overexpression of catalytically inactive PLD2 [21]. Elevated PLD2 activity has been found in various cancers including breast, colon, and kidney [2,22,23]. MDA-MB-231 breast cancer cells with high levels of PLD2 activity invade Matrigel in *in vitro* experiments, however, breast cancer cells with very low PLD2 activity, such as MCF-7, are less invasive [19]. Elevated PLD2 activity has been implicated in increased protease secretion, a hallmark of invasive cancer cells. Specifically, overexpression of PLD2 in mouse fibroblasts caused an increase in MMP-9 secretion; a further increase in MMP-9 secretion was observed upon stimulation with a PLD2 agonist [2].

Epidermal growth factor receptor (EGFR) is a transmembrane protein tyrosine kinase that belongs to the ErbB family of receptor protein kinases [24]. EGFR-mediated signaling is dysregulated in many epithelial cancers, promoting tumor growth and progression [25,26]. Accordingly, EGFR antagonists are used therapeutically to treat cancer [27]. EGFR has been implicated in the regulation of PLD2 activity [17,28]. For example, PLD2 is constitutively associated with EGFR in HEK 293 cells [17,26]. Stimulation of EGFR with EGF leads to stimulation of PLD2 activity [11,28,29,30]. Further, stimulation with EGF induces tyrosine phosphorylation of PLD2 [28]; the functional significance of this phosphorylation is still under investigation.

PLD2 plays a critical role in many cellular processes such as cell growth, cell survival, cell proliferation, apoptosis, cell migration, and adhesion [1,2,3,4]. Dysregulation of many of these processes is critical for progression of normal cells to cells with a malignant phenotype. Although existing data suggest that PLD2 plays a role in many stages of tumorigenesis, including transformation, progression, and invasion, the exact molecular mechanisms are not well understood. Previous studies from our lab have shown that overexpression of catalytically active PLD2 in EL4 thymoma cells lacking PLD2 results in increased spreading and elongation of transfected cells, while inactive PLD2 produces the opposite effect [15]. In the same study, cells expressing active PLD2 form more tumors in syngeneic mice, as compared to parental cells or cells expressing inactive PLD2. Since EL4 cells express little or no PLD2, these results suggest that inactive PLD2 may interfere with signal transduction through non-productive protein-protein interactions, independent of inhibition of endogenous PLD2 activity. The effects of PLD2 expression on growth factor-initiated signaling were not examined previously. In the current study, we utilize EL4 cells expressing PLD2 to test whether EGFR signal transduction is altered by expression of PLD2. Our results show that EL4 cells expressing active PLD2 have increased expression of EGFR and exhibit enhanced response to EGF with respect to proliferation and invasion.

## 2. Results and Discussion

### 2.1. Expression of EGFR in EL4 Lymphoma Cells

Whether EGFR is expressed in lymphomas has not been reported. We therefore tested for EGFR expression in parental and transfected EL4 cell lines that we have described previously [15]. To summarize, these cell lines are stably transfected with either active or inactive hemagglutinin-taged human PLD2. Parental (V7) cells express undetectable levels of PLD activity, while cells transfected with active PLD2 (C5) exhibit moderate levels of PLD activity as detected in intact cells and in membrane preparations. The expression level of the inactive PLD2 protein is higher than that of the active PLD2 protein in these clonal cell lines. 

**Figure 1:**
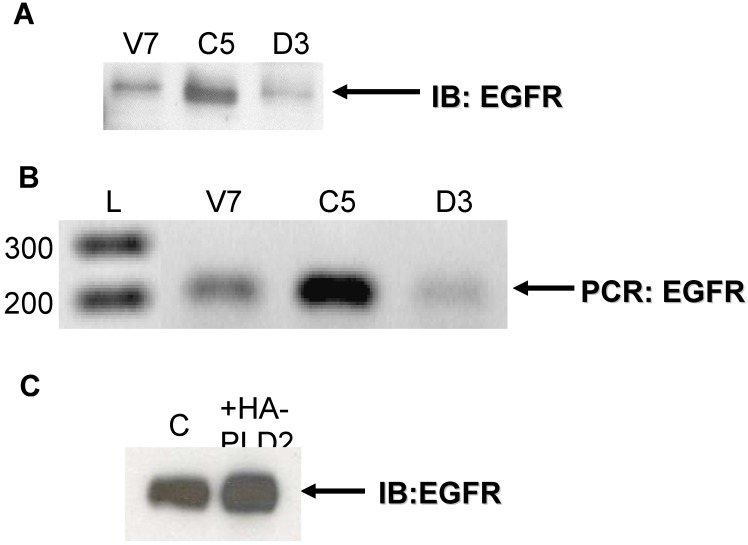
Expression of EGFR in EL4 and OVCAR3 cells. (a) Protein from EL4 whole-cell lysates (100 µg) was resolved by SDS-PAGE and transferred to a PVDF membrane. An antibody recognizing EGFR was used to detect EGFR expression. The blot was developed using chemiluminescence reagents. The arrow indicates EGFR. (b) Equal amounts of total RNA from each EL4 cell line were used to synthesize cDNA primed by anti-sense EGFR primer. PCR was performed using cDNA and murine EGFR primers in the linear range of amplification. PCR products were separated on an agarose gel, stained with ethidium bromide, and imaged. The arrow indicates PCR-amplified product from murine EGFR primers. L = DNA ladder (bp). (c) OVCAR3 cells were transiently transfected with or without HA-tagged PLD2 for 48 hours. Following transfection, cells were grown in serum-containing medium for 48 hours. Cells were then harvested and whole-cell extracts prepared. Extracts, equalized for protein, were resolved by SDS-PAGE and transferred to a PVDF membrane. An antibody recognizing EGFR was used to detect EGFR expression. The blot was developed using chemiluminescence reagents. The arrow indicates EGFR.

Equal amounts of protein lysates from V7 (parental), C5 (V7 + catalytically active PLD2), and D3 (V7 + catalytically inactive PLD2) cells were subjected to immunoblotting with anti-EGFR antibody. The results show that all cells express EGFR protein (Figure 1A). Notably, levels of EGFR protein are increased in cells expressing active PLD2 (C5) as compared to parental V7 cells and cells expressing inactive PLD2 (D3) (Figure 1A).

Next, we assessed EGFR mRNA expression in EL4 cells. Forward and reverse primers specific for murine EGFR were used in a polymerase chain reaction (PCR) to amplify cDNA from V7, C5, and D3 cells at a cycle number in the linear range for amplification of EGFR. The results (Figure 1B) show that all cell lines express EGFR mRNA (Figure 1B). Basal levels of EGFR mRNA are increased in cells expressing active PLD2 (C5) as compared to parental cells (V7) and cells expressing inactive PLD2 (D3).

In order to ensure that the PCR product amplified using EGFR primers was not another member of the EGFR family, the product was excised from the gel, cloned, and sequenced at the Genomics Core facility at Washington State University. The sequencing data was used in a nucleotide BLAST search. The excised PCR product was found to be 99% identical to both murine EGFR transcript variants 1 and 2 mRNA (data not shown) [31]. The results show that indeed the amplified PCR product is EGFR and not another member of the EGFR family. Hence, over-expression of PLD2 leads to increased levels of basal EGFR expression.

### 2.2. Effects of Over-Expression of PLD2 in OVCAR3 Ovarian Cancer Cells

Our results indicate that expression of PLD2 in EL4 cells leads to an increase in basal levels of EGFR protein and mRNA. To verify this result and also to ensure that results seen in Figure 1 are not exclusive to EL4 cells, we transiently over-expressed PLD2 in OVCAR3 cells (human ovarian cancer). Equal amounts of whole-cell lysates were immunoblotted for EGFR protein. The results show that OVCAR3 cells over-expressing HA-tagged PLD2 have higher basal levels of EGFR protein as compared to control cells (Figure 1C). Overall, results from Figure 1 indicate that over-expression of PLD2 results in increased expression of EGFR in a manner independent of cell type.

### 2.3. Effects of EGF Treatment on EL4 Cell Proliferation

**Figure 2:**
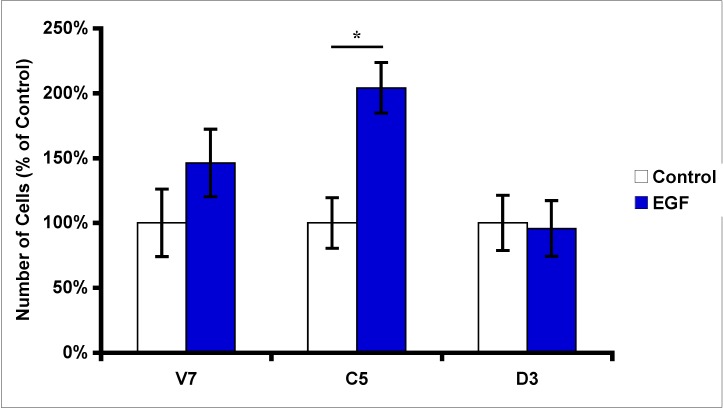
Effects of EGF treatment on EL4 cell proliferation. Equal numbers of EL4 cells (2 × 10^5^) were plated and grown overnight. Cells were then serum starved for 24 hours. Serum-starved cells were treated with 10 nM EGF for 24 hours. Following treatment, viable cells were counted using hemacytometer. Cell numbers are presented as percent of control (without serum); each data point represents mean ± SD from three separate wells. Statistical significance was determined using t-test: * p < 0.05.

Since over-expression of PLD2 results in increased expression of EGFR, the next question asked was whether this increase in basal EGFR was physiologically significant. To address the function of EGFR in EL4 cells, we treated serum-starved V7, C5, and D3 cells with 10 nM EGF, and then assessed EL4 cell proliferation after 24 hours. Our results (Figure 2) indicate that EGF induces a significant increase in proliferation of C5 cells over-expressing catalytically active PLD2. However, there was no significant increase in cell proliferation in response to EGF in parental cells (V7), or in cells expressing catalytically inactive PLD2 (Figure 2). Overall, these results indicate that over-expression of PLD2 leads to increase in cell proliferation upon EGF stimulation. These results also indicate that the EGFR is functional in EL4 cells expressing PLD2.

### 2.4. Effects of Protein Kinase Inhibitors on EGF-Induced C5 Cell Proliferation

**Figure 3:**
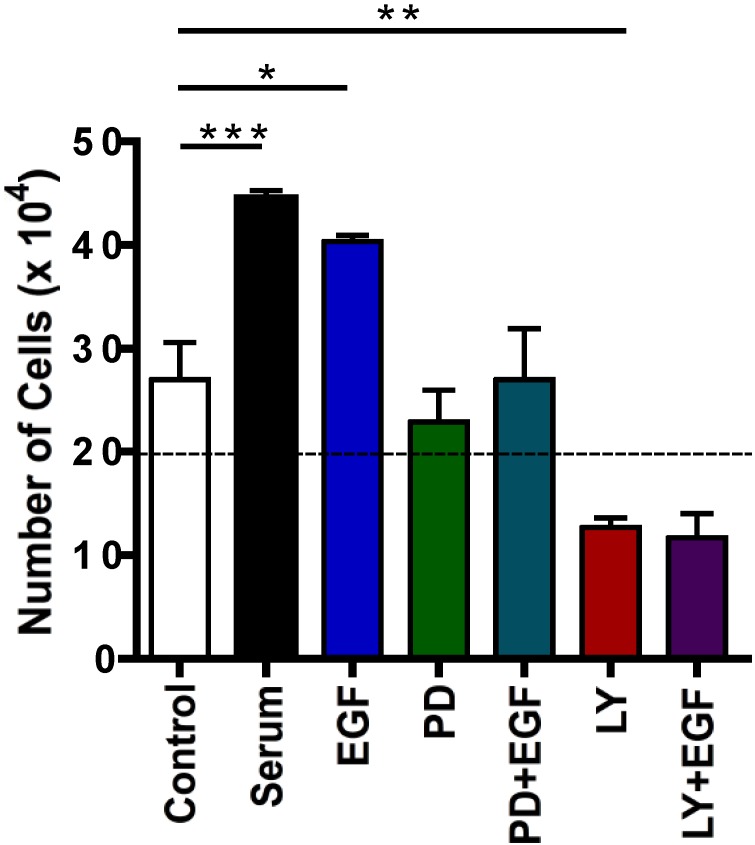
Effects of inhibitors on EGF-induced proliferation in C5 cells. Equal numbers of C5 cells (2 × 10^5^) were plated and grown overnight. Cells were serum starved for 24 hours, then incubated for 24 hours in triplicate with and/or without 10% FBS (positive control), 10 nM EGF, 2 pM PD15878 (“PD”: EGFR inhibitor), or 25 μM LY290042 (“LY”; PI3K inhibitor). Following treatment, viable cells were counted. The dashed horizontal line indicates the number of cells originally plated. Each data point represents mean ± SD for three wells of cells. Statistical significance was determined using t-test: * p < 0.05; ** p < 0.02; *** p < 0.01.

EGFR activation leads to the recruitment and activation of downstream targets facilitating cell growth. In particular, EGF activates the PI3K/Akt pathway leading to cell proliferation. In order to determine if the PI3K/Akt pathway is involved in the EGF-induced increase in proliferation in C5 cells, we treated C5 cells with or without various kinase inhibitors in the presence or absence of 10 nM EGF for 24 hours (Figure 3). Our results indicate that treatment with an EGFR kinase inhibitor, PD15878, inhibits EGF-induced C5 proliferation, as expected (Figure 3). The EGFR inhibitor also reduces basal proliferation, suggesting a broader role for EGFR in EL4 cells. In contrast, treatment with a PI3K inhibitor, LY290042, reduces both basal and EGF-induced C5 proliferation below control levels (Figure 3). This latter result suggests that LY294002, at the dose used, caused death of a portion of the serum-starved cells. This conclusion was confirmed by a time course study (data not shown), in which the number of viable cells was decreased by prolonged treatment of serum-starved cells with 25 μM LY294002. Overall, results from the inhibitor studies indicate that the PI3K/Akt pathway is necessary for viability, and thus for EGF-induced proliferation, in EL4 cells expressing PLD2. 

### 2.5. Time Course of EGF-Induced Akt Activation in EL4 Cells

**Figure 4:**
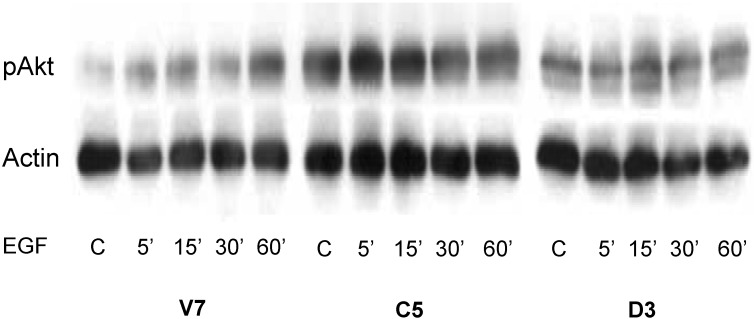
Time course of the effects of EGF on Akt activation in EL4 cell lines. Cells were serum starved 24 hours prior to EGF treatment. Serum-starved cells were treated for indicated times with or without (“C”) 10 nM EGF. Equalized protein (100 μg) from whole-cell lysates was resolved by SDS-PAGE and transferred to a PVDF membrane. An antibody recognizing phospho-Akt was used to detect activated Akt. Anti-actin antibody was used as a loading control. The blot was developed using chemiluminescence reagents.

In order to further investigate the role of the PI3K/Akt pathway, we examined the time course for Akt activation upon EGF treatment in all three EL4 cell lines. We treated the cells with 10 nM EGF from 0 to 60 minutes. Whole cell lysates were immunoblotted for phospho-Akt. The results (Figure 4) show that C5 cells with catalytically active PLD2 have higher basal phospho-Akt as compared to parental V7 cells and cells expressing catalytically inactive PLD2 (D3), confirming our previous findings (~ 2-fold increase) [15]. Further, EGF stimulation leads to a time-dependent increase in phospho-Akt levels only in V7 and C5 cells, and not in D3 cells (Figure 4). The level of EGF-stimulated Akt phosphorylation is higher in C5 as compared to V7 cells. Overall, cells expressing catalytically active PLD2 have increased levels of basal phospho-Akt, as well as EGF-induced phospho-Akt. In contrast, catalytically inactive PLD2 suppresses EGF-mediated Akt activation.

### 2.6. Effects of EGF-Induced Cell Invasion in EL4 Cells

**Figure 5:**
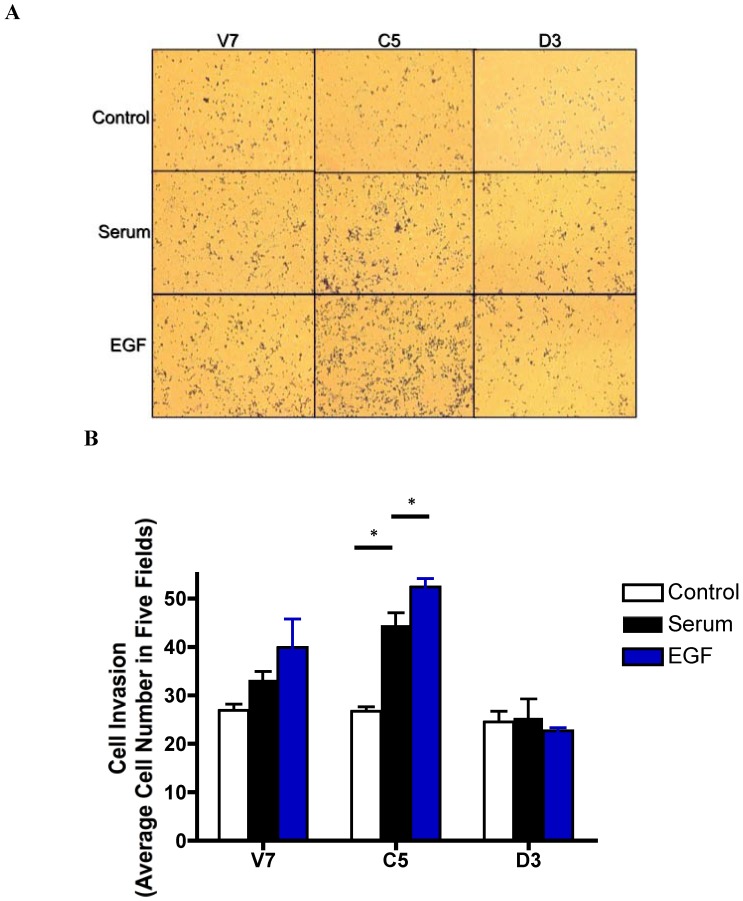
Effects of EGF treatment on EL4 cell invasion. Equal numbers (1 × 10^5^) of EL4 cells were plated in the upper chamber of 8-micron trans-well insert coated with 20 μg Matrigel. To the bottom chamber the following chemoattractants were added in serum-free medium: Control, 0.1% BSA; Serum, 10% FBS; EGF, 10 nM EGF. (a) After 24 hours, invaded cells were stained with Diff-Quik, photographed. (b) Migrated cells were quantified by cell counting. Data are plotted as percent of BSA control; each data point represents mean ± SD for three wells of cells. Statistical significance was determined using t-test: * p < 0.05.

Previous studies from our lab have shown that over-expression of catalytically active PLD2 results in increased spreading and elongation of transfected cells [15]. These are morphological changes that are characteristic of invasive cells. We therefore investigated the effects of EGF on invasion of EL4 cells expressing active or inactive PLD2. We used a modified Boyden chamber assay to assess EL4 cell invasion. Equal numbers of cells were added to the upper chamber of the trans-well inserts coated with Matrigel. To the bottom chamber, we added serum-free medium (negative control), serum-containing medium (positive control), or 10 nM EGF in serum-free medium. The results (Figure 5) show that C5 cells over-expressing catalytic active PLD2 display significantly increased cell invasion in response to serum and EGF as compared to V7 cells. V7 cells also display an increase in invasion in response to serum and EGF, but to a lesser extent (Figure 5). In contrast, no invasion was observed in response to either serum or EGF in D3 cells expressing catalytically-inactive PLD2.

### 2.7. Discussion

This study presents a new relationship between PLD2 and EGFR. Previous studies have shown that EGFR stimulates PLD2 activity, and that PLD2 and EGFR co-localize [17,28]. We for the first time show that over-expression of PLD2 leads to an increase in EGFR expression. This is also the first study that we are aware of that shows EGFR expression in a lymphoma cell line. EGFR receptors and response have been widely studied in cancer cells, and particularly in carcinoma cells. The EGFR is critically involved in cross-talk between different types of receptors [32]. Thus, EGFR expression in a lymphoma cell line is not necessarily surprising, but represents an under-explored aspect of lymphoma cell biology. In this study, we also demonstrate that PLD2 enhances EGF response in EL4 cells. 

Our group has generated and characterized multiple EL4 cell lines based on their response to PMA [32]. V7 cells are PMA-resistant, and do not activate Erk efficiently in response to PMA, unlike PMA-sensitive cells [33]. The difference in PMA sensitivity is due to a lack of RasGRP expression in PMA-resistant cells [34]. In order to validate our initial result of PLD2-induced increased basal EGFR expression, we transiently over-expressed PLD2 in OVCAR3 cells, which have been used in our laboratory previously. Our results (Figure 1C) indicate that a PLD2-induced increase in EGFR expression is not exclusive to EL4 cells.

We verified the functionality of the EGFR in EL4 cells via cell proliferation assays. Our results indicate that EL4 cells proliferate in response to EGF (Figure 2). These results demonstrate that indeed the EGFR in EL4 cells is functional and responds to EGF treatment. Further, our results indicated that EL4 cells expressing catalytically active PLD2 have a significant increase in EGF-induced proliferation compared to parental cells and cells expressing catalytically inactive PLD2 (Figure 2). This result maybe due in part to the fact that C5 cells have approximately two-fold higher expression of EGFR compared to V7 and D3 cells, providing increased EGFR to be activated by EGF. However, it is also likely that the presence of PLD2 enhances EGF response. This latter conclusion is supported by the negative effects of catalytically-inactive PLD2 on EGF response (Figure 4 and Figure 5). The relative contributions of EGFR up-regulation and enhanced EGFR signaling remain to be delineated. The mechanism(s) by which PLD2 can increase EGFR levels is not yet known. We hypothesize that, as a binding partner for EGFR [17,26], PLD2 may decrease the rate of EGFR down-regulation.

EGF-induced proliferation involves activation of downstream pathways such as the PI3K/Akt pathway. We investigated the role of this pathway in EGF-induced proliferation of EL4 cells. Our results indicate that the PI3K/Akt pathway is critical in EGF-induced EL4 cell proliferation (Figure 3); this is at least in part because the pathway is required for cell survival. Our signal transduction results (Figure 4) further support the fact that the PI3K/Akt pathway plays an important role in C5 cells in response to EGF response. Based on Figure 3 and Figure 4, we conclude that EGF-induced proliferation in C5 cells is dependent on a functional PI3K/Akt pathway. Others have demonstrated that PLD2 can modulate activation of the PI3K/Akt pathway [35], consistent with our results. However, the prominent role of PLD2 in EGF-mediated Akt activation is novel to the current study.

Our study establishes that EGF induces invasion in EL4 cells, and that cells expressing catalytically active PLD2 demonstrate the greatest increase in cell invasion upon EGF stimulation (Figure 5). These results support results from our laboratory and others demonstrating that PLD2 modulates various cellular events in a manner that can promote tumor progression [4,15]. In conclusion, our study presents a new role of PLD2 with respect to EGFR, a target of various anti-cancer drugs. PLD2 enhances EGFR expression and EGF responses in a manner that leads to increased mitogenesis and invasion.

## 3. Experimental Section

### 3.1. Materials

EGF was purchased from Sigma (St. Louis, MO, USA). PD158780, and LY294002 were purchased from Calbiochem (San Diego, CA, USA). 

### 3.2. Cell Culture

Clonal EL4 and OVCAR3 cells were maintained in RPMI-1640 (GIBCO BRL, CA, USA) supplemented with 10% fetal bovine serum (FBS) (Atlanta Biologicals, Atlanta, GA, USA) as previously described [15]. All cells were grown in 5% CO_2_/95% air at 37 °C on standard tissue culture dishes. Stably transfected EL4 cells were maintained in the presence of 0.25 mg/mL G418 (Calbiochem), except when cells were used for experiments, when they were maintained in medium without G418 for 24 hours prior to experiments.

### 3.3. Immunoblotting

Following treatment, cells were rinsed with 1X PBS, and harvested by scraping. Cells were collected by centrifugation at 1,200 x g. Whole cell lysates were prepared by lysing cells in lysis buffer containing 20 mM Tris (pH 7.4), 1% Triton X-100, 150 mM NaCl, 1 mM EGTA, 30 mM sodium pyrophosphate, 100 μM sodium orthovanadate, 1 mM phenylmethylsulfonyl fluoride, 10 μg/mL aprotinin, and 10 μg/mL leupeptin. Coomassie blue reagent (Pierce Chemical, Rockford, IL, USA) was used to determine protein concentrations. Equal amounts of protein (100 μg) were loaded in each lane of a 10% polyacrylamide gel, separated by SDS-PAGE, and transferred to PVDF membranes. Membranes were incubated with primary antibodies and developed using enhanced chemiluminescence reagents (Amersham Pharmacia Biotech, Piscataway, NJ, USA). Anti-EGFR, anti-phosho-Akt, and anti-Actin antibodies were from Cell Signaling Technology (Beverly, MA, USA). Blots were quantified using NIH Image software. 

### 3.4. Semi-Quantitative PCR

Total RNA was extracted using the RNeasy Mini kit from Qiagen. Genomic DNA was removed from total RNA by treating with DNase 1 from Invitrogen. Concentration and purity of total RNA were determined by absorbance at 260 nm and by OD_260/280_ ratio, respectively. Equal amounts (2 μg) of total RNA were used for cDNA synthesis primed by an EGFR anti-sense primer [36] using the Thermoscript kit according to the manufacturer’s instructions (Invitrogen). PCR was performed using equal amounts of cDNA and EGFR primers in a MyiQ cycler (BioRad) using PCR kit (Invitrogen) according to the manufacturer’s instructions. The linear range of PCR amplification for EGFR and 18s RNA was determined as described previously [37]. In order to confirm that the PCR primers amplified EGFR-specific mRNA only, the PCR product was excised from the agarose gel, purified, cloned, and sequenced. The sequencing data were used in a nucleotide BLAST search and compared to murine EGFR mRNA.

### 3.5. PLD2 Transfection

OVCAR3 cells were transiently transfected with a human HA-PLD2 construct [15] using Lipofectamine 2000 (Invitrogen) in OPTI-MEM media for 48 hours according to manufacturer’s instructions. Following transfection, cells were grown in serum-containing media for 48 hours. 

### 3.6. Cell Proliferation

Equal numbers (2 × 10^5^) of cells from each cell line were grown in 6-well plates in triplicates in serum-containing media for 24 hours. Prior to treatment, cells were serum starved overnight. Following treatment with growth factors and inhibitors, cells were collected by scraping, and viable cells were counted using Trypan blue staining. 

### 3.7. Cell Invasion Assay

*In vitro* cell invasion was measured using a modified Boyden chamber method (BD Biosciences, San Jose, CA). To the upper wells of the chamber, cells overexpressing active and inactive PLD2 and parental cells were added at a concentration of 100,000 cells in serum-free media. To the lower wells, media with 10% FBS was added. Invasion was measured as the ability of cells to pass through Matrigel (BD Biosciences), an artificial extracellular matrix that was used to coat the upper well. The membranes were fixed and stained with the Diff-Quik® dye kit. Cells that invaded into the lower wells were counted using a microscope.

### 3.8. Statistical Analysis

All experiments were repeated; representative results from three independent experiments are shown. For quantified data, values were expressed as mean ° SD of values obtained. Statistical significance was assessed by Student’s t-test, using InStat software (GraphPad).

## 4. Conclusions

This study has established that PLD2 up-regulates EGFR expression, as well as EGF-induced Akt activation, in a murine lymphoma cell line. These results reveal new aspects of the relationship between EGFR and PLD2, and provide insight into the therapeutic efficacy of EGFR and Akt protein kinase inhibitors in suppressing cancer cell proliferation and migration.
